# Supplemental *L*-Arginine Improves the Embryonic Intestine Development and Microbial Succession in a Chick Embryo Model

**DOI:** 10.3389/fnut.2021.692305

**Published:** 2021-10-06

**Authors:** Dong Dai, Hai-jun Zhang, Kai Qiu, Guang-hai Qi, Jing Wang, Shu-geng Wu

**Affiliations:** Laboratory of Quality and Safety Risk Assessment for Animal Products on Feed Hazards (Beijing) of the Ministry of Agriculture and Rural Affairs, Institute of Feed Research, Chinese Academy of Agricultural Sciences, Beijing, China

**Keywords:** chick embryo, intestinal development, *L-arginine*, microbial succession, prenatal nutrition

## Abstract

Early colonization of intestinal microbiota plays an important role in intestinal development. However, the microbial succession at an embryonic stage and its assembly patterns induced by prenatal nutrition are unknown. In the present study, we used a chick embryo model to investigate the effects of *in ovo* feeding (IOF) of *L*-arginine (Arg) on the intestinal development and microbial succession of embryos. A total of 216 fertile eggs were randomly distributed into 2 groups including the non-injected control group and IOF of Arg group with 7 mg/egg. The results showed that IOF Arg increased the intestinal index, absolute weight of jejunum, and improved jejunal morphology in terms of villus width and surface area (*p* < 0.05). The relative mRNA expressions of *mTOR* and *4E-BP1* were up-regulated and accompanied by higher contents of Mucin-2 in the Arg group (*p* < 0.05). There was a significant elevation in contents of serum glucose and high-density lipoprotein cholesterol, whereas there was a decreased low-density lipoprotein cholesterol in the Arg group (*p* < 0.05). Additionally, Proteobacteria and Firmicutes were major intestinal bacteria species at the embryonic stage. However, Arg supplementation targeted to shape assembly patterns of microbial succession and then changed microbial composition (*p* = 0.05). Meanwhile, several short-chain fatty acids (SCFAs)-producing bacteria, such as *Roseburia, Blautia*, and *Ruminococcus* were identified as biomarkers in the Arg group (LDA > 3, *p* < 0.05). Accordingly, significant elevated concentrations of SCFAs, including lactic acid and formic acid, were observed in the Arg group (*p* < 0.05), accompanied by the higher concentration of butyric acid (0.05 < *p* < 0.10). In conclusion, prenatal Arg supplementation improved embryonic intestine development by regulating glucose and lipid homeostasis to supply more energy for chick embryos. The possible mechanism could be the roles of Arg in shaping the microbial assembly pattern and succession of the embryonic intestine, particularly the enrichment of potential probiotics. These findings may contribute to exploring nutritional strategies to establish health-promoting microbiota by manipulating prenatal host-microbe interactions for the healthy development of neonates.

## Introduction

Early intestinal development has the potential to be a determinant for the lifelong health of a host. Numerous evidence suggest that the microbial community is an essential driver of intestinal innate immune programming (1, 2) and plays critical roles in the differentiation and maturation of epithelial cells (3, 4), maintenance of intestinal barrier, and absorption (5, 6). Consequently, the initial stage of microbial colonization is regarded as a focus on modulating intestinal health to avoid poor nutrient availability, pathogen infection, and higher mortality in early postnatal life (7, 8). Owing to resilience and stability in the microbial ecosystem (9, 10), clarifying the early colonization characteristics of microbiota is crucial to explore appropriate intervention methods. It was previously thought that chick embryos were sterile and that the initial intestinal microbiota originated from a post-hatch environment (11). However, recent studies have reported the presence of diverse microbes in chick embryos using 16S rRNA sequencing technology (12–15). Nevertheless, there is little information concerning the effects of an exogenous intervention on embryonic microbiota at present.

Considering the plasticity and transitivity of early microbiota, the prenatal nutrition intervention may alter the colonization of embryonic microbes and can further shape neonatal intestinal microbiota more effectively (8). Compared with mammals during the perinatal and lactation period, the development of chick embryos and microbial colonization are out of the strong control of maternal effects during hatching (16), which can be easily observed and manipulated *in vitro*. Therefore, the chick embryo system could be used as an ideal model to study interactions between host development and microbial colonization at the embryonic stage (7, 9, 17). Meanwhile, the last few days pre-hatch is the most critical period for the intestinal development in chicks (18). Taken all together, the later embryonic stage may be considered as the best window period (19) so that the employ of *in ovo* feeding (IOF) method is optimal and practicable to determine the effects of prenatal nutrient intervention on intestinal development and microbial colonization in a relatively separate system (7).

As an essential amino acid for gestating mammals and developing chicks, *L*-arginine (Arg) is crucial for intestinal development and neonatal growth (20, 21). However, birds are unable to synthesize Arg as lacking carbamyl phosphate and dihydropyrrole-5-carboxylate synthase involved in the urea cycle (22). Previous studies have indicated that IOF of Arg improves intestinal health and development of post-hatch chickens by multiple mechanisms, including suppressing the *iNOS* methylation, activating *mTOR* signaling pathway, and regulating energy metabolism (23–25). Further, our previous study found that the intestinal microbiota participates in the improvement of intestinal development induced by IOF of Arg in post-hatch chicks (7). We speculated that the microbial colonization and succession at the embryonic stage may be driven by IOF of Arg. Nevertheless, the microbial succession of the embryonic gut and its assembly patterns induced by prenatal Arg supply are still unknown. These are hindered by considerable knowledge gaps in the origin and colonization of intestinal microbiota (13).

The purpose of this study was to investigate the effects of prenatal Arg intervention on intestinal development and serum biochemical parameters of chick embryos. In addition, characteristic changes in colonization and succession of embryonic microbiota induced by IOF of Arg were clarified. Our findings may provide new insight into the roles of IOF of Arg in shaping succession and establishing health-promoting intestinal microbiota at the embryonic stage and advance our understanding of the fundamental knowledge about prenatal host-microbe interactions.

## Materials and Methods

### Treatment Solutions and IOF Procedures

Fertile eggs (56.42 ± 0.42 g) aged 31 weeks from White Leghorn layers were incubated in the automatic-controlled incubator (Chengdu Beili Agricultural Technology Co., Ltd. Chengdu, China). The temperature in the incubator was kept at 37.8°C and the relative humidity at 60% according to standard hatchery procedures. IOF of Arg was performed at the age of embryos 17.5 (17.5 E). A total of 216 fertile eggs with similar weight were selected (*p* > 0.05) and randomly distributed into 2 groups with 6 replicates. Unlike the non-injected control group (NC), the IOF of Arg group (Arg) was injected with 0.1 ml saline containing 7 mg Arg. The 21-gauge needle was used to insert Arg into the amniotic fluid. The optimal concentration of Arg injection solution was chosen from the preliminary study of our team (7). Briefly, Arg solutions were freshly prepared and kept in the incubator for 2 h before injection. To avoid subsequent contamination, the surrounding environment and eggs were disinfected with 75% ethanol before the IOF.

### Sample Collection

At the embryotic age of 17 (17 E, pre-IOF), 19 (19 E, 1.5 d after IOF), and 21 (21 E, 3 d after IOF and the day of birds are about to hatch), a chick embryo with average egg weight from each replicate was selected for sampling. Blood samples were collected into tubes without anticoagulants and then centrifuged at 3,000 × g for 15 min at 4°C to obtain serum and stored at −80°C. Duodenum, jejunum and ileum were collected and weighed to calculate the intestinal index (intestinal weight/embryonic weight × 100%). Then about 1 cm segments of the middle of jejunum and ileum were collected and fixed in 10% neutral-buffered formalin for morphological measurements. The remaining intestinal segments and cecum were also collected and snap-frozen in liquid nitrogen, and stored at −80°C for further analysis.

### Intestinal Morphology

After wash, dehydration, clarification, and paraffin embedding procedures for intestinal segments, serial sections were then cut at 2 μm and placed on glass slides to be deparaffinized in xylene, rehydrated, and stained with hematoxylin and eosin. The villus height (VH, from the tip of the villus to the crypt opening) and villus width (VW, the length of the diameter perpendicular to the villus height) were determined by light microscopy (BX51, Olympus Co., Tokyo, Japan). Additionally, the surface area (SA) of villus was calculated according to the formula 2π × VH × (VW/2) (26).

### Quantitative Real-Time PCR Analysis

Total RNA was extracted from jejunal tissues with the TRIzol reagent (Tiangen Biotech Co., Ltd, Beijing, China Beijing, China) following the standard procedure. The quality and purity of RNA were assessed with Epoch Microplate Spectrophotometer (BioTek Instruments, Inc., VT, USA). The cDNA was obtained by reverse transcription of the total RNA with the first-strand synthesis kit (Tiangen Biotech Co. Ltd., Beijing, China Beijing, China). Real-time quantitative PCR reactions were performed in duplicate using SYBR Green on an ABI 6 flex real-time PCR instrument (Thermo Fisher Scientific, MA, USA). The Supplementary Table 1 showed primers sequences of *mTOR, 4E-BP1, S6K1*, and *18S rRNA* used in this study. Relative mRNA expression levels were calculated according to the 2^−ΔΔCt^ method (27).

### Measurement of Jejunal Secretory Immunoglobulin a (SIgA) and Mucin-2 Contents

The mucosa was weighed and homogenized in an ice-cold 0.85% NaCl solution in a chilled homogenizer and immediately centrifuged at 3,500 × g for 10 min at 4°C to obtain the supernatant fluids. Then, SIgA and Mucin-2 contents were immediately assayed using the chicken enzyme-linked immunosorbent assay kit (Shanghai Enzyme-linked Biotechnology Co., Ltd., Shanghai, China) according to the protocol of the manufacturer. SIgA and Mucin-2 contents were calculated from the standard curve and expressed as ng per ml.

### Serum Biochemical Parameters

Serum glucose (GLU), triglycerides (TG), total cholesterol (TC), high-density lipoprotein cholesterol (HDL-C), and low-density lipoprotein cholesterol (LDL-C) concentrations were measured using the KHB400 automatic biochemical analyzer (Kehua Bioengineering, Co. Ltd., Shanghai, China).

### Short-Chain Fatty Acid (SCFAs) Profiles

Frozen cecal digesta samples were thawed at 4°C and diluted 5-fold with sterile PBS in sterile screw-cap tubes before being homogenized and centrifuged at 12,000 rpm for 10 min at 4°C. The concentrations of lactic acid, formic acid, acetic acid, propionic acid, and butyric acid were detected using a gas chromatograph (GC-2010 ATF, Shimadzu, Japan) equipped with a capillary column (30 m × 0.25 mm × 0.5 μm). The nitrogen was supplied at a flow rate of 18 ml/min as a carrier gas (12.5 MPa). The temperature of the injector and detector was 180°C. The initial oven temperature was 80°C, which was then increased to 170°C at a rate of 5°C/min. The concentration was calculated using the raw data multiplied by the dilution ratio.

### DNA Extraction and PCR Amplification of 16S rRNA Gene Sequences

Microbial DNA was extracted from intestinal samples of chick embryos at 17 E, 19 E, and 21 E, using the E.Z.N.A Soil DNA Kit (Omega Bio-tek, Norcross, GA, USA) according to the instructions of the manufacturer. The V3-V4 regions of bacterial 16S rRNA gene were amplified using primer 338F (5′-ACTCCTACGGGAGGCAGCA-3′) and 806R (5′-ACTCCTACGGGAGGCAGCA-3′). Purified amplicons were pooled in equal amounts and pair-end sequenced (2 × 250 bp). Throughput analysis was performed at Shanghai Personal Biotechnology Co., Ltd., using the Illumina MiSeq platform. Sequences were processed and taxonomy assigned using Quantitative Insights into Microbial Ecology 2 (QIIME 2) (28). Amplicon sequence variants (ASVs) were determined with Dada2 using the denoise-paired method. Classification of ASVs at various taxonomic levels was implemented using the Greengenes database. Shared and unique species between groups were used to generate a Venn diagram. β-diversity was estimated using principal coordinate analysis (PCoA) accompanied by the analysis of similarities (ANOSIM) to assess the significance of microbial community differences among groups. Before linear discriminant analysis (LDA) combined effect size (LEfSe) was employed (LDA > 3, *p* < 0.05), Welch's *t*-test was employed to explore the differences in the relative abundances of bacteria (29). The co-occurrence of microbial communities was analyzed at the genus level. Significant Spearman correlations (*R* > 0.5, *p* < 0.05) among top 50 genus were noted based on the relative abundances. Visualization of the co-occurrence network was conducted using a Python package NetworkX. Spearman correlation analysis was conducted on the potential relationship between intestinal microbiota and phenotypes. The raw sequencing data have been deposited into the NCBI Sequence Read Archive database (accession number: PRJNA705406).

### Statistical Analysis

Data analysis was performed using SAS Version 9.2 (SAS Institute Inc., Cary, NC, USA). The *t*-test was used to measure the effects of treatment. Differences were considered statistically significant at *p* < 0.05, and a tendency toward significance considered at 0.05 ≤ *p* < 0.10.

## Results

### Hatching Parameters Concerning Embryo Development and Hatchability

The hatching parameters including the absolute weight of embryo and intestine, index of the intestine, and hatchability are shown in Table 1. As can be seen, there were no differences in the absolute weight of the embryo and total intestine at 19 E and 21 E (*p* > 0.05). Simultaneously, significant differences in the index of total intestine and hatchability were not found between NC and Arg groups (*p* > 0.05). However, the greater absolute weight and index of jejunum were observed in the Arg group than those in the NC group at 21 E (*p* = 0.031, *p* = 0.021, respectively).

**Table 1:** Effects of *in ovo* feeding of *L*-arginine on embryo development.

**Item**	**NC** ^**1**^	**Arg** ^**2**^	***P*-value**
**The age of embryos 19**			
Embryo weight, g	36.45 ± 1.52	37.87 ± 2.40	0.180
Absolute weight of total intestine, g	0.48 ± 0.11	0.54 ± 0.11	0.291
Index of total intestine, %	1.23 ± 0.20	1.43 ± 0.28	0.152
**The age of embryos 21**			
Embryo weight, g	40.04 ± 1.87	39.21 ± 1.07	0.294
Absolute weight of duodenum, g	0.24 ± 0.03	0.26 ± 0.05	0.255
Index of duodenum, %	0.60 ± 0.09	0.67 ± 0.13	0.212
Absolute weight of jejunum, g	0.23 ± 0.04^b^	0.29 ± 0.06^a^	0.031
Index of jejunum, %	0.56 ± 0.09^b^	0.73 ± 0.16^a^	0.021
Absolute weight of ileum, g	0.25 ± 0.04	0.23 ± 0.10	0.672
Index of ileum, %	0.62 ± 0.10	0.59 ± 0.25	0.745
Absolute weight of total intestine, g	0.71 ± 0.07	0.78 ± 0.16	0.286
Index of total intestine, %	1.78 ± 0.17	1.99 ± 0.39	0.196
Hatchability, %	89.05 ± 5.98	88.10 ± 3.52	0.792

a,b *Means within a row with no common superscripts differ significantly (p < 0.05)*.

1 *NC, non-injected control group*;

2 *Arg, in ovo of feeding of 7 mg L-arginine group. Intestinal index, % = intestine weight/embryo weight × 100. Data are represented with the means ± standard deviation*.

### The Development of Embryonic Intestine

**Figure 1:**
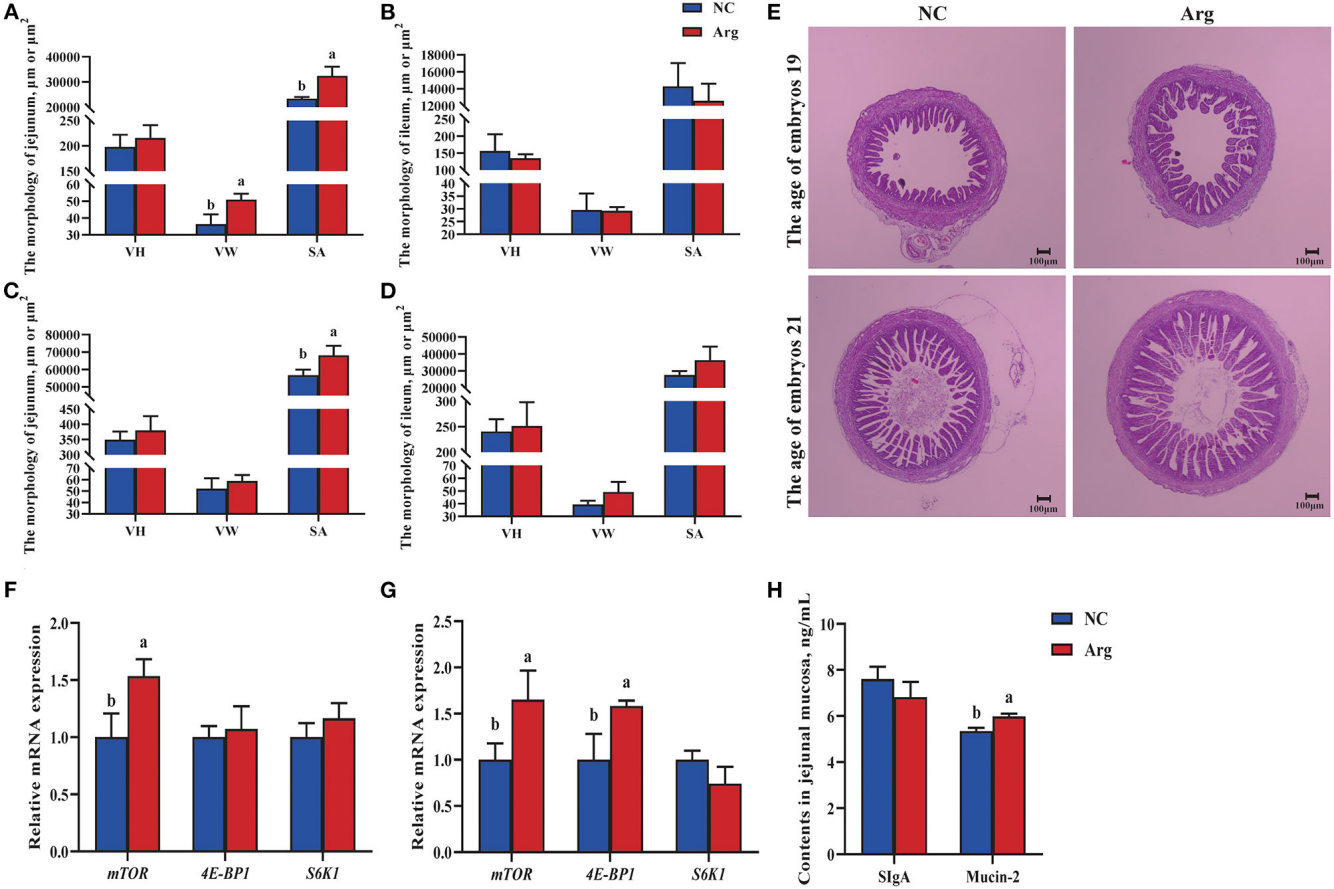
Effects of *in ovo* feeding of *L*-arginineon the development of embryonic intestine of chicks. ^a−b^Values at the same index with no common superscripts differ significantly (*p* < 0.05). **(A,B)** The intestinal morphology of jejunum and ileum at the age of embryos 19 (19 E), respectively; **(C,D)** The intestinal morphology jejunum and ileum at the age of embryos 21 (21 E), respectively; **(E)** Intestinal morphological structure of jejunum at 19 and 21 E, respectively. The pictures were observed at 40 × magnification; **(F,G)**, the relative mRNA expression of genes in the *mTOR* pathway of jejunum at 19 E and 21 E, respectively; **(H)**, the contents of SIgA and Mucin-2 of jejunum at 21 E; NC, non-injected control group; Arg, *in ovo* of feeding of 7 mg *L*-arginine group; VH, villus height; VW, villus width; SA, surface area of villus, which was calculated according to the formula 2π × VH × (VW/2); SIgA, Secretory Immunoglobulin A.

Figure 1 shows the alterations in the development of the embryonic intestine concerning the morphology, expressions of *mTOR* pathway, and contents of SIgA and Mucin-2 between the NC and Arg groups. Compared with the NC group, the increased VW of jejunum was observed in Arg group at 19 E along with the higher SA of jejunum at 19 E and 21 E (*p* < 0.05) (Figures 1A,C). However, there were no significant differences in the morphology of ileum between NC and Arg groups at 19 E and 21 E (*p* > 0.05) (Figures 1A,C). Therefore, only the jejunum was used to study the effects of IOF of *L*-Arg on gene expressions of the *mTOR* pathway and contents of SIgA and Mucin-2 (Figures 1F–H). Compared with the NC group, the relative mRNA expression of *mTOR* was up-regulated in the embryonic jejunum of the Arg group at 19 E (*p* < 0.05). Similarly, the up-regulated relative mRNA expressions of *mTOR* and *4E-BP1* were also found, accompanied by the higher content of Mucin-2 in the Arg group at 21 E (*p* < 0.05). However, no significant differences in the content of SIgA were observed (*p* > 0.05).

### Serum Biochemical Parameters

**Figure 2:**
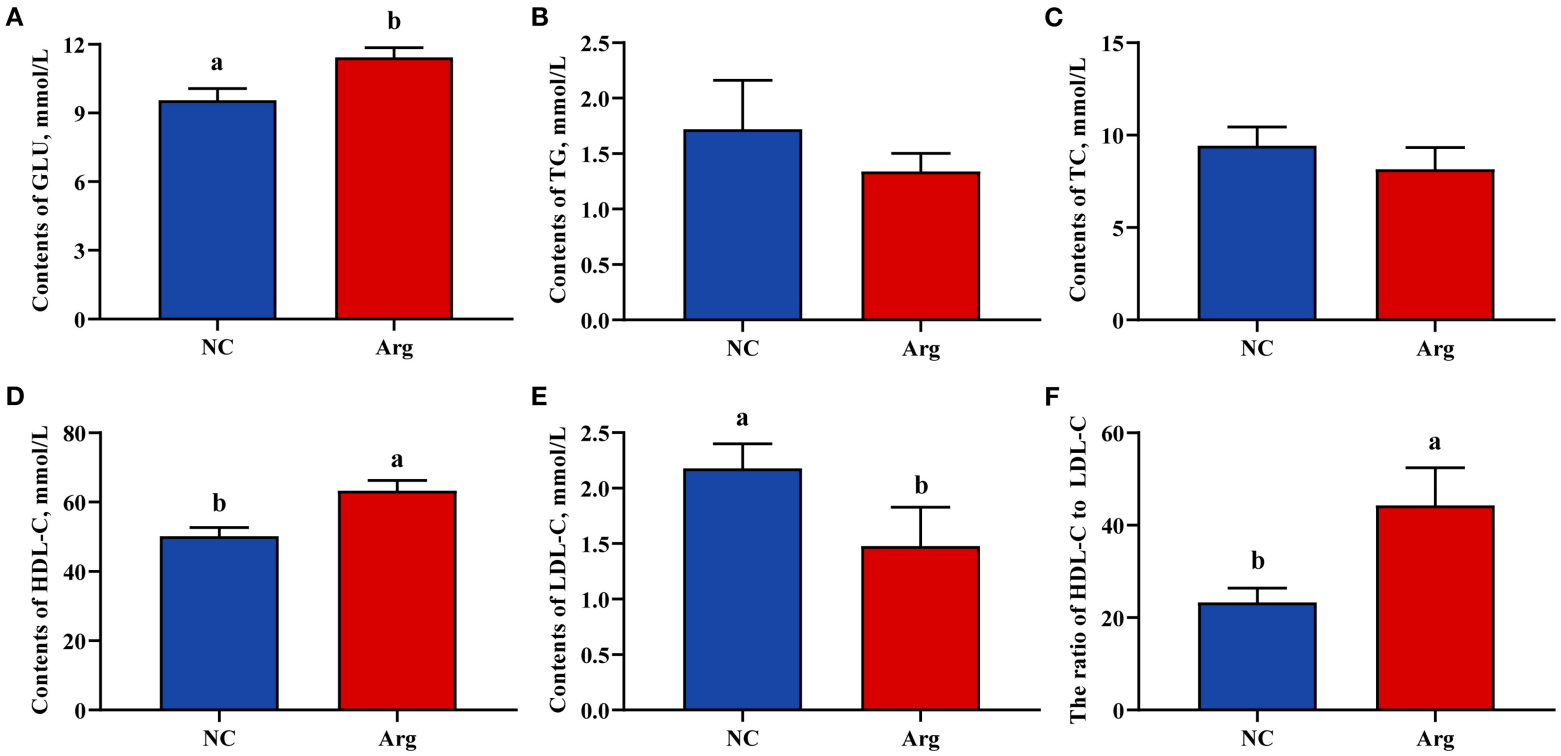
Effects of *in ovo* feeding of *L*-arginine on the serum biochemical parameters at the age of embryos 21. **(A)** Glucose contents; **(B)** Triglycerides contents; **(C)** Total cholesterol contents; **(D)** High-density lipoprotein cholesterol contents; **(E)** Low-density lipoprotein cholesterol contents; **(F)** The ratio of high-density lipoprotein cholesterol to low-density lipoprotein cholesterol; ^a,b^Values at the same index with no common superscripts differ significantly (*p* < 0.05). NC, non-injected control group; Arg, *in ovo* of feeding of 7 mg *L*-arginine group; GLU, glucose; TG, triglycerides; TC, total cholesterol; HDL-C, high-density lipoprotein cholesterol; LDL-C, low-density lipoprotein cholesterol.

The effects of IOF of Arg on serum biochemical indicators of chicks including the contents of GLU, TG, TC, HDL-C, and LDL-C are shown in Figure 2. The higher content of GLU was observed in the Arg group than that in NC group at 21 E (*p* < 0.05). Moreover, we also found that the content of HDL-C was higher and was accompanied by a decreased content of LDL-C in the Arg group than that in the NC group (*p* < 0.05). Thus, the ratio of HDL-C to LDL-C in the Arg group was significantly higher than that of the NC group (*p* < 0.05). However, there were no significant effects on the contents of TG and TC of chicks at 21 E (*p* > 0.05).

### The Embryonic Intestinal Microbiota Analysis

**Figure 3:**
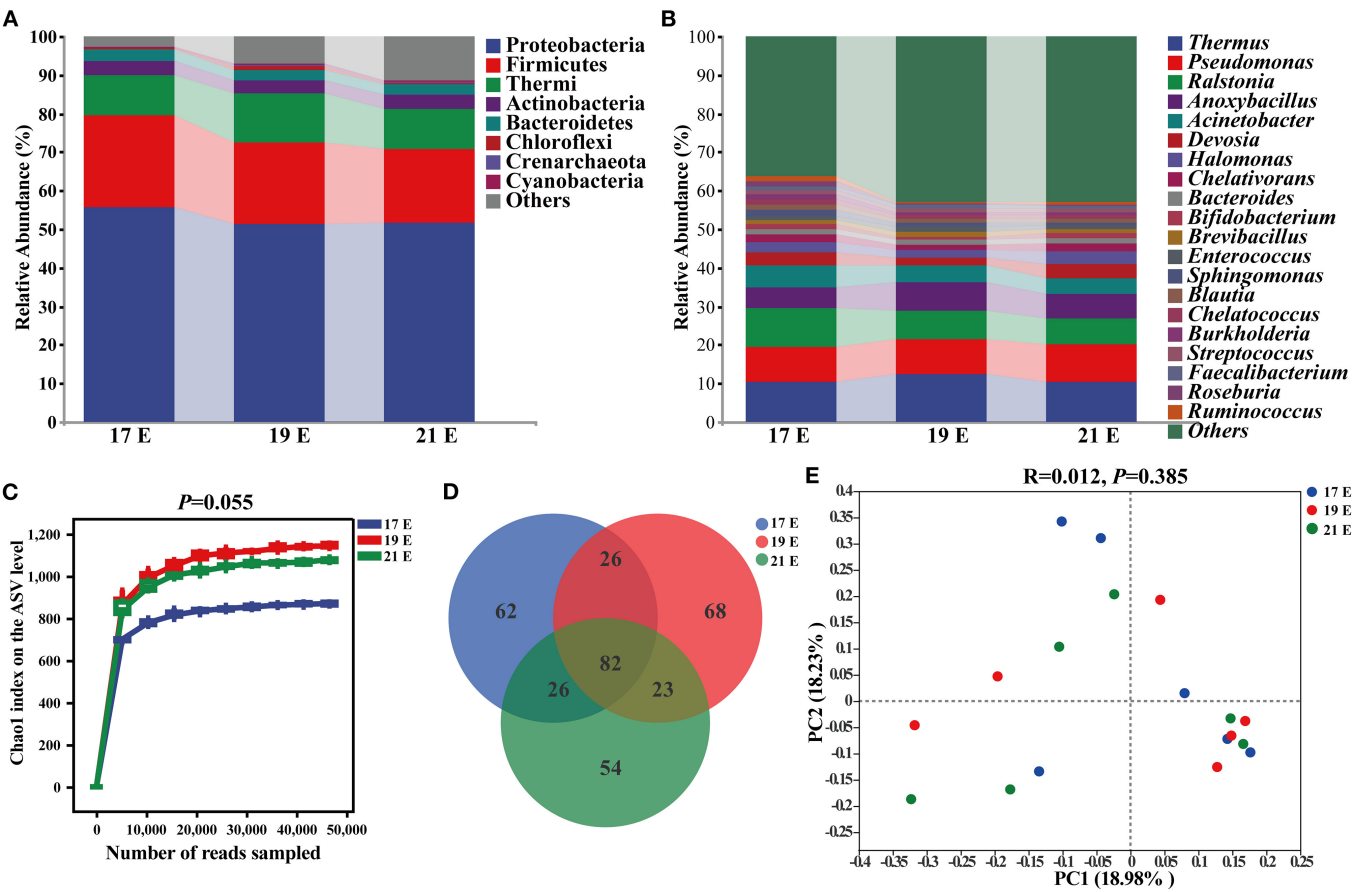
Composition and diversity analysis of intestinal microbiota from control chick embryos at the age of embryos 17–21. **(A,B)** At phylum and genus level, respectively; **(C)** Chao1 index on the ASVs level; **(D)** A Venn diagram based on genus level; **(E)** Principal coordinate analysis (PCoA) based on weighted unifrac distance; 17, 19, and 21 E, at the age of embryos 17, 19 and 21.

In order to study the establishment and succession of microbiota at the embryonic stage, we compared the microbial composition and abundance of the NC group at 17 E, 19 E, and 21 E (Figure 3). The ASV level of each group increased sharply before reaching a plateau, which indicated that the number of microbial sequences represented the microbial communities well as the rarefaction curves tended toward saturation (Figure 3C). There was an increasing tendency in the α-diversity of the intestinal microbiota at 19 E and 21 E than that at 17 E (*p* = 0.055). The major intestinal microbiota species in phylum were Proteobacteria (51.33–55.85%), Firmicutes (19.22–23.59%), Thermi (10.50–12.56%), Actinobacteria (3.39–3.57%), Bacteroidetes (2.62–3.07%), and Chloroflexi (0.31–0.89%) at 17 E, 19 E, and 21 E (Figure 3A). Although there was similarity of microbial species, a decreasing proportion of Proteobacteria, Firmicutes, and Bacteroidetes were observed at 19 E and 21 E compared with the 17 E. The dominant genus across all groups were *Thermus, Pseudomonas, Ralstonia, Anoxybacillus, Acinetobacter, Pelagibacterium*, and *Halomonas* which together, contributed >44% of the whole genus (Figure 3B). Moreover, the Venn diagram demonstrated that the genus differed and there were 62, 68, and 54 specific genus at 17 E, 19 E, and 21 E, respectively. In addition, a total of 65 shared genera were identified among 3 groups that had been colonizing at an embryonic late stage (Figure 3D). These were defined as generalists. However, PCoA based on weighted uniFrac distance did not reveal a separation of microbiota among 3 groups (*R* = 0.012, *p* = 0.385) (Figure 3E)

**Figure 4:**
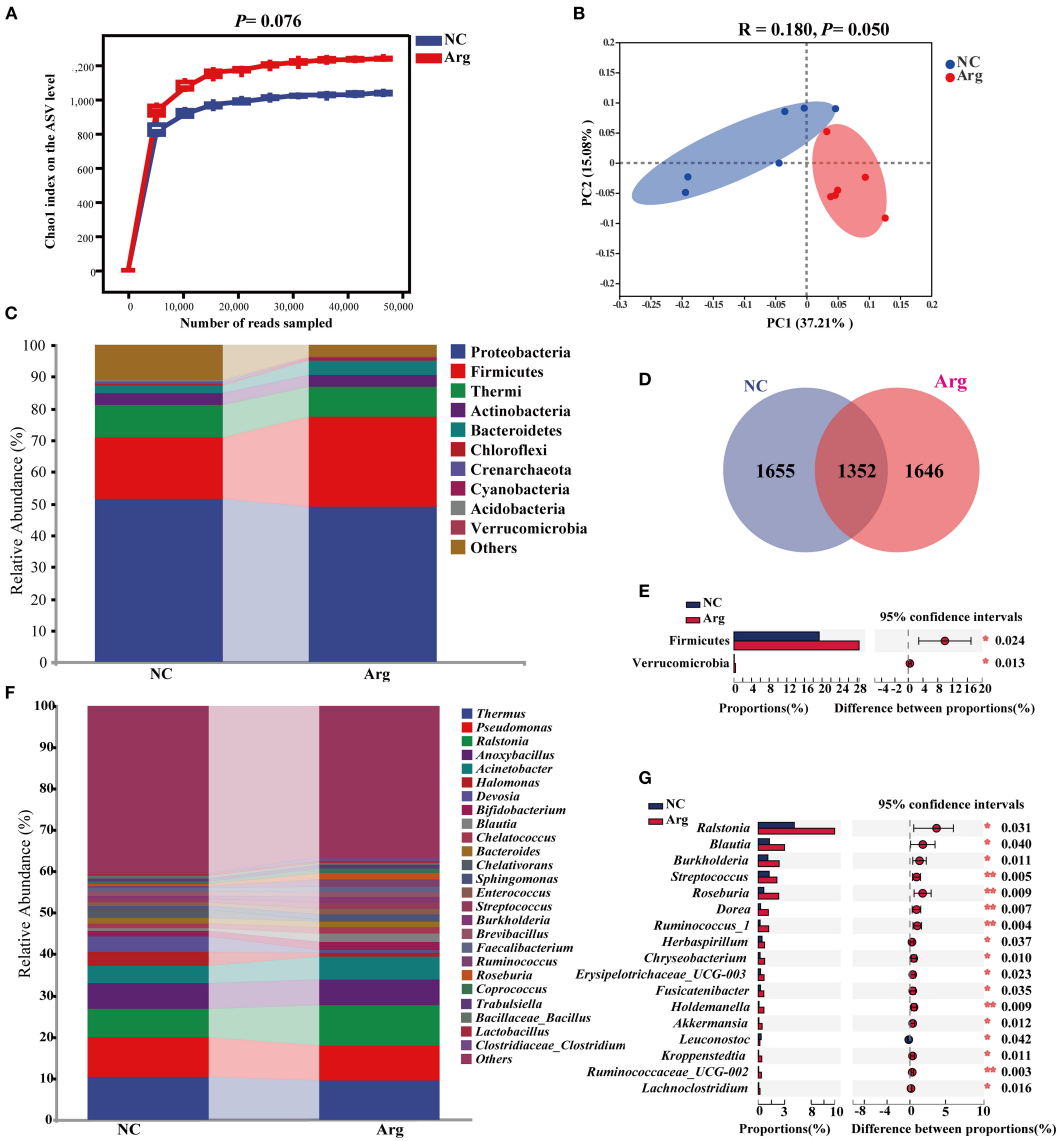
Effects of *in ovo* feeding of *L*-arginine on composition and diversity analysis of intestinal microbiota from chick embryos at the age of embryos 21. **(A)** Chao 1 index on ASVs level; **(B)** Principal coordinate analysis (PCoA) based on weighted unifrac distance; **(D)** A Venn diagram based on ASVs level; **(C,F)** The composition of intestinal microbiota at phylum and genus level, respectively; **(E,G)** differential species identified at phylum and genus level, respectively; NC, non-injected control group; Arg, injected with 7 mg *L*-arginine group.

To investigate the difference induced by IOF of Arg in embryonic microbiota, the intestinal microbiota compositions of the Arg group were also analyzed at 19 E and 21 E (Figure 4). Similarly, the rarefaction curves of the Arg group increased sharply and tended toward saturation (Figure 4A). An increasing tendency in the Chao1 index of the Arg group was observed compared with the NC group at 21 E (*p* = 0.076). However, no significant difference was observed at 19 E (*p* > 0.05) (Supplementary Figure 1). β-diversity analysis was performed to compare the overall microbial profiles between the NC and Arg groups. As can be seen from Figure 4B, samples from different groups occupied distinct positions at 21 E (COMP1, 37.21%; COMP2, 15.08%). ANOSIM analysis also suggested that the compositions of microbiota were dissimilar between 2 groups at 21 E (*R* = 0.180, *p* = 0.050). However, we failed to observe a significant difference in intestinal microbiota between NC and Arg groups at 19 E (*p* > 0.05) (Supplementary Figure 1). The Venn diagram revealed the difference in ASVs, and there were 1,655 and 1,646 unique ASVs in NC and Arg groups, respectively (Figure 4D). Taxonomic compositions were analyzed at phylum and genus levels (Figures 4C,F). Although the species of the dominated phylum and genus did not change compared with the NC group, there was a significant difference in the abundances of phylum and genus at 21 E. A higher abundance of Firmicutes (19.36%:28.41%) with a lower abundance of Proteobacteria (51.20%:48.72%) were observed in the Arg group at 21 E.

**Figure 5:**
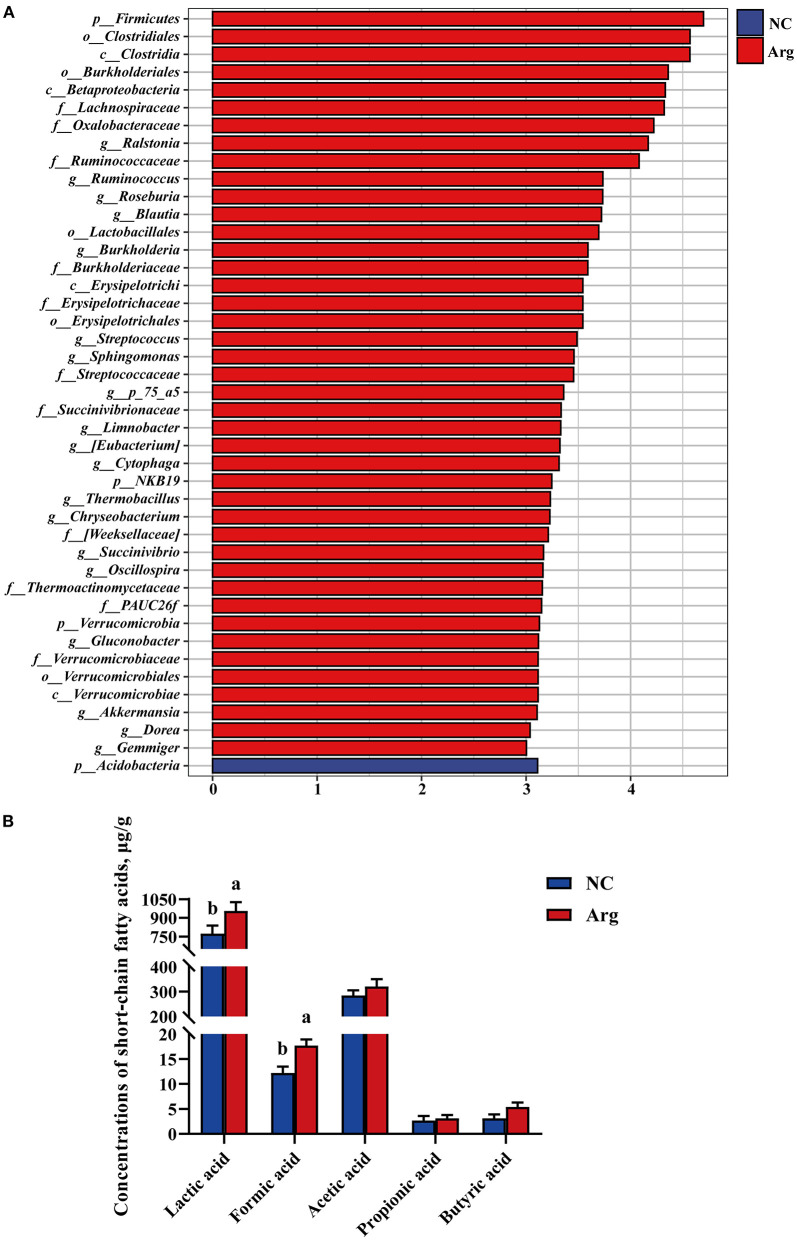
Linear discriminant analysis effect size and effects of *in ovo* feeding of *L*-arginine on concentrations of cecal short-chain fatty acids at the age of embryos 21. **(A)** Species with significant difference that have an LDA score greater than the estimated value 3. And the length of the histogram represents the LDA score; **(B)** Concentrations of cecal short-chain fatty acids. ^a,b^Values at the same index with no common superscripts differ significantly (*p* < 0.05); NC, non-injected control group; Arg, injected with 7 mg *L*-arginine group.

Moreover, IOF of Arg significantly increased the abundance of Firmicutes and Verrucomicrobia at 21 E (*p* < 0.05) (Figure 4E). At the genus level, the abundance of *Ralstonia, Blautia, Burkholderia, Streptococcus, Roseburia, Dorea, Ruminococcus_1, Herbaspirillum, Chryseobacterium, Erysipelotrichaceae_UCG-003, Fusicatenibacter, Holdemanella, Akkermansia, Kroppenstedtia, Ruminococcaceae_UCG-002*, and *Lachnoclostridium* were higher in the Arg group than that in the NC group (*p* < 0.05) (Figure 4G). The LEfSe analysis was further performed to identify bacteria as biomarkers for entire microbiota from phylum to genus. As shown in Figure 5A, the intestinal microbiota in the Arg group was enriched with Lachnospiraceae (*Roseburia, Blautia* and *Dorea*), Ruminococcaceae (*Ruminococcus, Gemmiger*, and *Oscillospira*), Streptococcaceae (*Streptococcus*), Burkholderiaceae (*Ralstonia, Burkholderia* and *Limnobacter*), Sphingomonadaceae (*Sphingomonas*), and Verrucomicrobiaceae (*Akkermansia*). However, Acidobacteria was observed to be enriched only in the NC group.

### The Embryonic Intestinal SCFA Profiles

Short-chain fatty acids, major carbohydrate fermentation products of gut microbiota serving as indicators of microbial activity, were detected and quantified here in Figure 5B. Compared with the NC group, the contents of lactic acid and formic acid were significantly increased in the Arg group (*p* < 0.05). Otherwise, a higher concentration of butyric acid was also observed in the Arg group (0.05 < *P* < 0.10). However, no significant differences in acetic acid and propionic acid profiles were observed between NC and Arg groups (*p* > 0.05).

### Microbial Communities Co-occurrence Network Analysis

**Figure 6:**
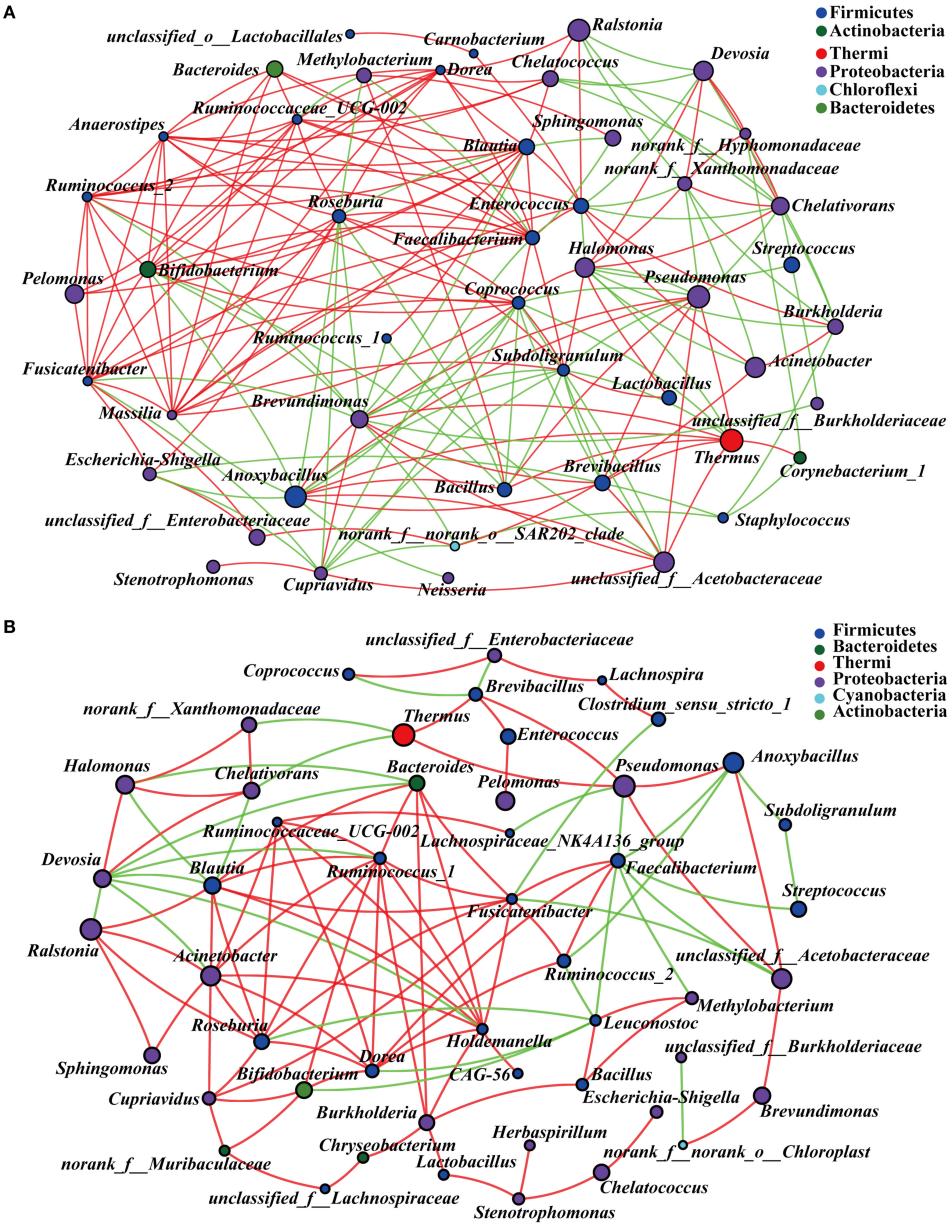
Microbial communities co-occurrence network analysis across the embryonic late stage (the age of embryos 17–21). **(A)** Intestinal microbiota from control chick embryos; **(B)** Intestinal microbiota from injected with 7 mg *L*-arginine group; The figure shows species with *p* < 0.05 based on the spearman's correlation. The size of nodes indicates the relative abundance of the species. Red and green lines represent positive and negative correlations between two nodes, respectively.

In order to explore the co-existence and interaction of species during intestinal microbiota succession, the co-occurrence of microbial communities across the embryonic late stage was analyzed. The correlation network analysis showed 193 and 109 significant positive or negative correlations in NC and Arg groups, respectively (Figure 6). The average path length between the two nodes was 2.31 edges with a diameter of 5 edges in the NC group, whereas the larger value of average path length and diameter were observed in the Arg group (3.62 and 11, respectively). All genus in the network were assigned to bacteria phyla. Proteobacteria (42.55%) and Firmicutes (46.94%) made up the largest proportions in NC and Arg groups, respectively. Based on degree centrality, namely, closeness centrality and betweenness centrality scores, the top 8 shared genus were selected as the keystone genus including *Enterococcus, Brevundimonas, Faecalibacterium, Anoxybacillus, Coprococcus, Roseburia, Subdoligranulum*, and *Blautia* in the NC group (Figure 6A). Likewise, *Fusicatenibacter, Ruminococcus_1, Holdemanella, Faecalibacterium, Dorea, Blautia, Bacteroides*, and *Roseburia* were selected as the keystone genus in the Arg group (Figure 6B). Maybe these microbes as keystone taxa played critical roles in the co-occurrence network. In addition, there is a large change in the proportion of positive and negative links (77.33%:22.67%) among keystone taxa in the Arg group compared with the NC group (58.12%:41.88%).

### Correlations Between Intestinal Microbiota and Phenotypes

**Figure 7:**
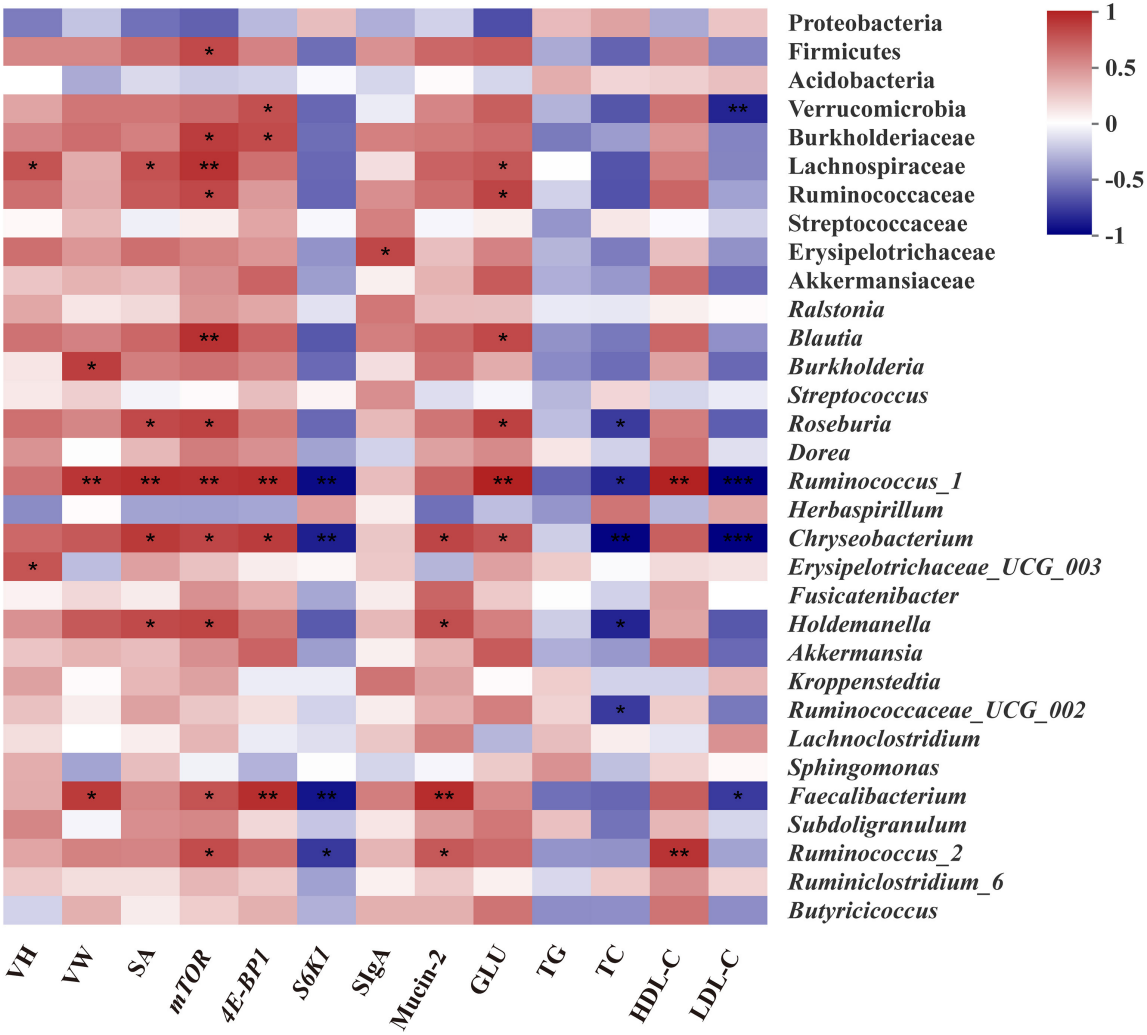
Spearman's correlation analysis between the abundances of intestinal microbiota and intestinal or serum biochemical parameters. Red represents a positive correlation and blue represents a negative correlation. Significant correlations are noted by.01 < *p* ≤ 0.05^*^, 0.001 < *p* ≤0.01^**^, *p* ≤0.001^***^. VH, Villus height; VW, Villus width; SA, Surface area; SIgA, Secretory Immunoglobulin A; GLU, Glucose; TG, Triglycerides; TC, Total cholesterol; HDL-C, High-density lipoprotein cholesterol; LDL-C, Low-density lipoprotein cholesterol.

The Spearman correlation analysis was employed to explore the differential abundance of bacteria associated with intestinal development and serum biochemical indicators (Figure 7). The abundance of family Lachnospiraceae, Ruminococcaceae, and Erysipelotrichaceae which are affiliated to phylum Firmicutes showed significant positive correlations (*p* < 0.05) with VH, SA, the expression of *mTOR*, the content of GLU, and SIgA, respectively. In addition, the significant positive correlation between the abundance of family Burkholderiaceae, and expressions of *mTOR* and *4E-BP1* were observed from the heatmap (*p* < 0.05). At the genus level, the abundance of *Blautia* and *Roseburia* belonging to the family Lachnospiraceae were positively associated with SA, the expression of *mTOR* and GLU concentration (*p* < 0.05). The abundance of *Ruminococcus_1, Ruminococcus_2*, and *Faecalibacterium*, belonging to the family Ruminococcaceae, were positively correlated with VW, SA, and expressions of *mTOR* and *4E-BP1*, Mucin-2, GLU, and HDL-C concentration, but negatively correlated with the content of TC and LDL-C and the expression of *S6K1* (*p* < 0.05), respectively. Moreover, the significant positive correlation between the abundance of Erysipelotrichaceae derivatives (*Erysipelotrichaceae_UCG-003* and *Holdemanella*) and VH, SA, the expression of *mTOR* and the content of Mucin-2 (*p* < 0.05), were also found.

## Discussion

Prenatal nutrition is involved in embryonic development and neonatal growth, realizing the potential to be the main determinant of lifelong health for the host (20). Accumulating evidence has demonstrated health-beneficial effects of IOF of Arg on post-hatch chicks in terms of improving digestive and immune barrier function, and enhancing growth performance (21, 23, 30). In the present study, the positive effects of IOF of Arg on the development of embryonic intestine were evidenced by increased absolute weight and index and improved intestinal morphology (including VW and SA) (Figure 1) which could enhance intestinal nutrient digestibility and absorption and may further improve subsequent growth performance of post-hatch chicks (7). These beneficial effects could be attributed to the activated *mTOR* signaling pathway by Arg supplementation increase epithelial cells protein synthesis to promote the development of embryonic intestine (31). Likewise, there were up-regulated relative mRNA expressions of *mTOR* and *4E-BP1* in this study, accompanied by the elevated content of Mucin-2 in the Arg group (Figure 1). Mucin-2 covered intestinal epithelial surface can be secreted by goblet cells and plays a crucial role in protecting the intestinal epithelial tissue from pathogen infection, which further indicated improved barrier integrity and immune homeostasis of the intestinal mucosa by IOF of Arg (32).

Otherwise, another effect was reported: that IOF of Arg could alter the energy metabolism of post-hatch chicks by improving liver gluconeogenesis and stimulating the release of insulin (25). Our previous study applied metabolomics to characterize the metabolite changes of post-hatch chicks induced by IOF of Arg in terms of galactose and lipid metabolisms (7). However, there was little information concerning the changes in the metabolic profiles at the embryonic stage, which contributed to further research on interactions between intestinal development and energy metabolism. The present study showed that IOF of Arg could increase the contents of serum GLU and HDL-C (Figure 2). In fact, the lipid utilization of chick embryos was constrained by the limited availability of oxygen at the later embryonic stage (25). HDL-C was identified as participating in the lipid translocation from peripheral tissues to the liver for bile acid secretion (33), which could further improve lipid metabolism (34). Simultaneously, the decreased serum LDL-C level and the elevated ratio of HDL-C to LDL-C in chick embryos also indicated the improvement of lipid metabolism (35). Additionally, chick embryo protein was compulsively mobilized for hepatic gluconeogenesis at the later embryonic stage due to the insufficient availability of energy and carbohydrates (25). Arg as the gluconeogenic precursor was not only converted into GLU (36), but also stimulated the release of hormones for regulating energy metabolism (37). Therefore, the improvement of intestinal development might also be attributed to the modulated energy metabolism, which directed more energy toward the rapid growth of the intestine at the embryonic stage (7, 25).

It was previously thought that the microbial colonization of chick intestine originated from post-hatch environment (13). Recent studies reported the presence of diverse microbes in chick embryos and suggested the microbial colonization in offspring embryos originate from the maternal oviduct (14–17). In the current study, the microbial composition and succession characteristics of the intestine at the embryonic stage were further clarified (Figure 6). In the control chicks, the embryonic intestine microbiota was predominantly composed of Proteobacteria and Firmicutes. In addition, Proteobacteria showed the highest abundance accounting for more than 50% of all species, which were consistent with previous studies in poultry (13). Additionally, Proteobacteria was also the dominant bacterial taxa in the embryonic intestine of humans and mammals without intraamniotic infection (38, 39), which suggested that bacterial colonization might be consistent across species. One hypothesis was that the high abundance of Proteobacteria in the intestines might drive the development of the intestinal immune system, not for permanent colonization (40).

Lipid aerobic oxidation of yolk was the dominant mean to supply energy for chick embryos (25). The extremely low carbohydrate metabolism might also explain the low abundance Firmicutes at the embryonic stage (41). In fact, microbial communities were not only a mere assembly of species individuals, but also a complex of interconnected ecological communities with communication, recombination, and coevolution (42). Hence, the co-existence and interaction during the process of embryonic microbiota assembly were analyzed with co-occurrence network methods in order to explore ecological drivers of microbiota succession. Notably, a total of 8 shared genus were further selected as the keystone genus to drive the microbial community succession in the NC group, suggesting non-random assembly patterns of intestinal microbiota in chick embryos. Interestingly, the genus belonging to Firmicutes showed a more symbiotic relationship, whereas a more competitive relationship was shown in the genus belonging to Proteobacteria, which were consistent with the previous research on soil microbial communities (43). We further discovered that more than 40% proportion of interactions were competitive and exploitative during the intestinal microbiota assembly, which might be owing to the sharp transition of metabolism and the limited energy supply in the later stage of chick embryos (44–46). As a result, the competitive interactions of dominant Proteobacteria might also disturb the balance of microbial ecosystems and subsequently posed negative effects on embryonic development (43, 47).

It is remarkable that prenatal Arg supplementation could improve the balance of microbial ecosystems by shaping patterns of assembly of embryonic gut microbiota in this study. Unlike the NC group, *Fusicatenibacter, Ruminococcus_1, Holdemanella, Dorea*, and *Blautia* were selected as the keystone genus in the Arg group to drive the microbial community succession (Figure 6B). There was a 19.21% increase in the proportion of the symbiotic relationship in the Arg group, which indicated interactions of microbe-microbe had been changed and further shaped the different structures of microbial communities (48, 49). It was evidenced by the results of the PCoA and differential species analysis (Figure 4B). However, the previous study discovered that not all embryonic microbes were transmitted to post-hatch chicks, and some “core microbes” that could permanently exist in the embryonic and post-hatch stage were identified (13). Our results showed that the abundance of 12 microbes belonging to “core microbes” were increased in the Arg group, including *Ralstonia, Blautia, Burkholderia, Streptococcus, Dorea, Sphingomonas, Ruminococcus, Chryseobacterium, Akkermansia, Faecalibacterium, Oscillospira*, and *Butyricicoccus* (13). Moreover, dynamic alterations that increased the abundance of Firmicutes at the expense of Proteobacteria in the Arg group were consistent with the early gut microbial succession in post-hatch chicks (7). Thus, these observations demonstrated that prenatal Arg supplementation targeted to shape microbial assembly patterns and then accelerated microbial succession and maturation in chick embryos toward the early microbiota in post-hatch chicks. Nevertheless, the biological mechanisms of the induced changes by supplemental Arg in the microbial succession remain unknown. One possible explanation was that prenatal Arg supplementation could change the microenvironment and modulate amino acids metabolism by intestinal bacteria (50, 51), which might, in turn, affect the abundance and activity of some special bacteria.

In fact, the prenatal establishment of a metabolically active microbiome is essential for the developing fetus, which favors differentiation, proliferation, and maturation of intestinal epithelial cells (3, 4, 52). The elevated concentrations of lactic acid, formic acid, and butyric acid further demonstrated that the prenatal intestine not only harbored the presence of microbiome but was also metabolically active (39). Next, in order to better understand the role of characteristic changes of microbial colonization in intestinal development, several species as biomarkers were identified in the Arg group. Lachnospiraceae, Ruminococcaceae, and Peptococcaceae as SCFAs-producing bacteria were enriched in the Arg group (53), which could be responsible for the elevated cecal SCFAs in chick embryos (Figure 5B). In particular, *Roseburia* could produce a significant amount of butyric acid to regulate energy generation and epithelium cells response, which could promote the intestinal development (54). Likewise, a positive correlation was also found between the abundance of *Roseburia* and the expression of *mTOR* and intestinal SA in the current study. Meanwhile, the increased lactic acid produced by *Streptococcus* could be sensed by G protein-coupled receptor (GPR) 81 on the paneth and stromal cells to accelerate intestinal stem cell (ISC)-mediated epithelial development in a Wnt3/β-catenin-dependent manner (55). Additionally, numerous studies identified that *Blautia, Faecalibacterium*, and *Ruminococcus* were associated with improvements in glucose and lipid homeostasis (56–58). The consistent results of high correlations with the concentrations of GLU, TC, H-DLC, and L-DLC were also observed in this study. In fact, as the fermentation product of *Blautia, Faecalibacterium*, and *Ruminococcus*, SCFAs can inhibit insulin signaling and fat accumulation to improve glucose and lipid homeostasis by activating the GPR41 and GPR43 (59, 60). Interestingly, the previous study reported that *Roseburia, Blautia, Faecalibacterium*, and *Ruminococcus* were identified to be core genus in the representative populations of the world, and there was, notably, a positive synergy and crosstalk with each other (54, 61, 62). In the current study, the crucial roles of symbiotic relationships among *Roseburia, Blautia, Faecalibacterium*, and *Ruminococcus* were also observed in shaping microbial communities as the keystone genus (Figure 6). Hence, these microbes may be targeted to improve intestinal development and microbial ecosystems as potential probiotics (55, 63, 64). Although the role of Burkholderiaceae in animal intestines remains unclear, an increased abundance of *Burkholderia* accompanied by improvements of intestinal structure and growth performance were observed in chickens fed with probiotics (65). Burkholderiaceae, in addition, showed highly positive correlations with the mRNA level of *mTOR* and *4E-BP1* in the present study, suggesting a benefit of *Burkholderia* for intestinal development (66). Moreover, *Akkermansia* also played a crucial role in promoting ISC-mediated epithelial development and stimulating Mucin-2 production (67), which might partly explain the increased Mucin-2 in our study. Consequently, *Akkermansia* had been regarded as a healthy biomarker of infant intestinal microbiota and development (68) and could be next-generation probiotics like *Lactobacillus* and *Bifidobacterium* (69). To sum it all up, the effects of prenatal Arg supplementation on intestinal development could be partially responsible for the capability to shape embryonic microbiota, particularly the enrichment of potential probiotics.

**Figure 8:**
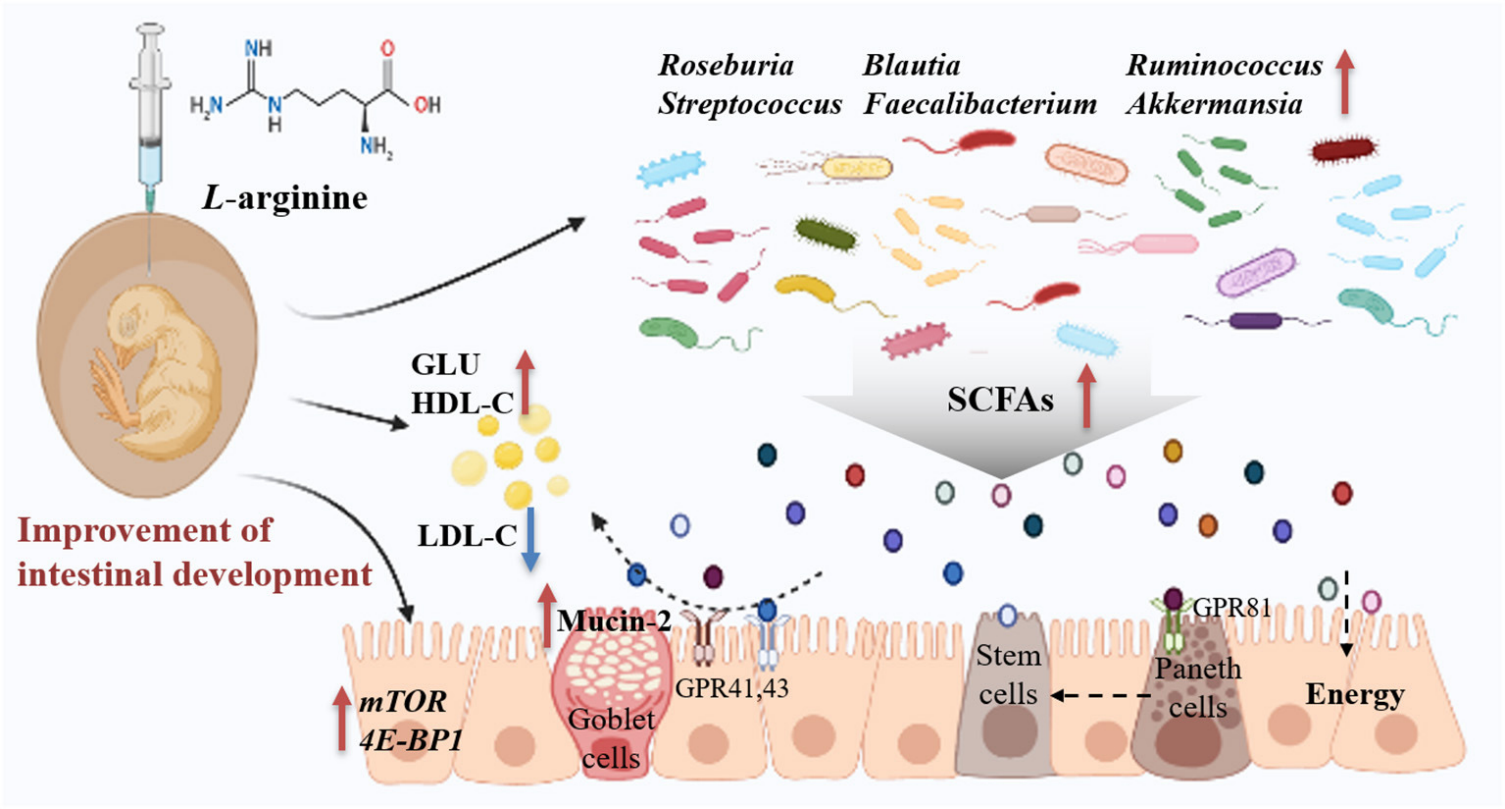
Summary of clarifying how prenatal *L*-arginine supplementation improves the embryonic intestine development in a chick embryo model. The red up-arrow represents upregulated, whereas the blue down-arrow represents downregulated; GLU, Glucose; GPR, G protein-coupled receptors; HDL-C, High-density lipoprotein cholesterol; LDL-C, Low-density lipoprotein cholesterol; SCFAs, short-chain fatty acids.

In summary, prenatal Arg supplementation improved embryonic intestine development by regulating glucose and lipid homeostasis to supply more energy for chick embryos. The possible mechanism could be the roles of Arg in shaping the microbial assembly pattern and succession at the embryonic stage, particularly in the enrichment of potential probiotics (Figure 8). These findings may contribute to exploring nutritional strategies to establish health-promoting microbiota by manipulating prenatal host-microbe interactions for the healthy development of neonates.

## Data Availability Statement

The datasets presented in this study can be found in online repositories. The names of the repository/repositories and accession number(s) can be found in the article/Supplementary Material.

## Ethics Statement

The animal study was reviewed and approved by Animal Care and Use Committee of the Feed Research Institute of Chinese Academy of Agricultural Sciences.

## Author Contributions

DD performed animal experiments, analyzed the data, and wrote the manuscript. JW and G-hQ contributed to the experimental design and the revision of the manuscript. HZ, KQ, and S-gW provided continuous guidance and assisted with the data analysis. All authors read and approved the manuscript.

## Funding

This study was supported by Beijing Natural Science Foundation (6214046), China Agriculture Research System (CARS-40-K12), Beijing Innovation Consortium of Agriculture Research System (BAIC04-2018), and the Agricultural Science and Technology Innovation Program (ASTIP) of the Chinese Academy of Agricultural Sciences.

## Conflict of Interest

The authors declare that the research was conducted in the absence of any commercial or financial relationships that could be construed as a potential conflict of interest.

## Publisher's Note

All claims expressed in this article are solely those of the authors and do not necessarily represent those of their affiliated organizations, or those of the publisher, the editors and the reviewers. Any product that may be evaluated in this article, or claim that may be made by its manufacturer, is not guaranteed or endorsed by the publisher.

## References

[1] Romano-Keeler J Moore DJ Wang C Brucker RM Fonnesbeck C Slaughter JC. Early life establishment of site-specific microbial communities in the gut. Gut Microbes. (2014) 5:192–201. 10.4161/gmic.28442 24637795 PMC4063844

[2] Kabat AM Srinivasan N Maloy KJ. Modulation of immune development and function by intestinal microbiota. Trends Immunol. (2014) 35:507–17. 10.1016/j.it.2014.07.010 25172617 PMC6485503

[3] Sommer F Bäckhed F. The gut microbiota–masters of host development and physiology. Nat Rev Microbiol. (2013) 11:227–38. 10.1038/nrmicro2974 23435359

[4] Ivanov II Atarashi K Manel N Brodie EL Shima T. Induction of intestinal Th17 cells by segmented filamentous bacteria. Cell. (2009) 139:485–98. 10.1016/j.cell.2009.09.033 19836068 PMC2796826

[5] Garrett WS Gordon JI Glimcher LH. Homeostasis and inflammation in the intestine. Cell. (2010) 140:859–70. 10.1016/j.cell.2010.01.023 20303876 PMC2845719

[6] Bäckhed F Manchester JK Semenkovich CF Gordon JI. Mechanisms underlyin? g the resistance to diet-induced obesity in germ-free mice. Proc Natl Acad Sci USA. (2007) 104:979–84. 10.1073/pnas.0605374104 17210919 PMC1764762

[7] Dai D Wu SG Zhang HJ Qi GH Wang J. Dynamic alterations in early intestinal development, microbiota and metabolome induced by in ovo feeding of L-arginine in a layer chick model. J Anim Sci Biotechnol. (2020) 11:19. 10.1186/s40104-020-0427-5 32175081 PMC7063725

[8] Jiang L Feng C Tao S Li N Zuo B Han D. Maternal imprinting of the neonatal microbiota colonization in intrauterine growth restricted piglets: a review. J Anim Sci Biotechnol. (2019) 10:88. 10.1186/s40104-019-0397-7 31737268 PMC6844051

[9] Rubio LA. Possibilities of early life programming in broiler chickens via intestinal microbiota modulation. Poult Sci. (2019) 98:695–706. 10.3382/ps/pey416 30247675

[10] Kogut MH. The effect of microbiome modulation on the intestinal health of poultry. Anim Feed Sci Technol. (2019) 250:32–40. 10.1016/j.anifeedsci.2018.10.008

[11] Wielen PW Keuzenkamp DA Lipman LJ Knapen F Biesterveld S. Spatial and temporal variation of the intestinal bacterial community in commercially raised broiler chickens during growth. Microb Ecol. (2002) 44:286–93. 10.1007/s00248-002-2015-y 12219265

[12] Lee S La TM Lee HJ Choi IS Song CS Park SY. Characterization of microbial communities in the chicken oviduct and the origin of chicken embryo gut microbiota. Sci Rep. (2019) 9:6838. 10.1038/s41598-019-43280-w 31048728 PMC6497628

[13] Ding J Dai R Yang L He C Xu K Liu S. Inheritance and establishment of gut microbiota in chickens. Front Microbiol. (2017) 8:1967. 10.3389/fmicb.2017.01967 29067020 PMC5641346

[14] Shterzer N Rothschild N Sbehat Y Stern E Nazarov A Mills E. Large overlap between the intestinal and reproductive tract microbiomes of chickens. Front Microbiol. (2020) 11:1508. 10.3389/fmicb.2020.01508 32760363 PMC7372964

[15] Akinyemi FT Ding J Zhou H Xu K He C Han C. Dynamic distribution of gut microbiota during embryonic development in chicken. Poult Sci. (2020) 99:5079–90. 10.1016/j.psj.2020.06.016 32988546 PMC7598139

[16] Milani C Duranti S Bottacini F Casey E Turroni F Mahony J. The first microbial colonizers of the human gut: composition, activities, and health implications of the infant gut microbiota. Microbiol Mol Biol Rev. (2017) 81:e00036-17. 10.1128/MMBR.00036-17 29118049 PMC5706746

[17] Arab JP Martin-Mateos RM Shah VH. Gut-liver axis, cirrhosis and portal hypertension: the chicken and the egg. Hepatol Int. (2018) 12:24–33. 10.1007/s12072-017-9798-x 28550391 PMC6876989

[18] Iji PA Saki A Tivey DR. Body and intestinal growth of broiler chicks on a commercial starter diet. 1. intestinal weight and mucosal development. Br Poult Sci. (2001) 42:505–13. 10.1080/00071660120073151 11572627

[19] Siwek M Slawinska A Stadnicka K Bogucka J Dunislawska A Bednarczyk M. Prebiotics and synbiotics - in ovo delivery for improved lifespan condition in chicken. BMC Vet Res. (2018) 14:402. 10.1186/s12917-018-1738-z 30558599 PMC6296066

[20] Wu G Bazer FW Cudd TA Meininger CJ Spencer TE. Maternal nutrition and fetal development. J Nutr. (2004) 134:2169–72. 10.1093/jn/134.9.2169 15333699

[21] Gao T Zhao MM Li YJ Zhang L Li JL Yu LL. Effects of in ovo feeding of L-arginine on the development of digestive organs, intestinal function and post-hatch performance of broiler embryos and hatchlings. J Anim Physiol Anim Nutr. (2018) 102:e166–75. 10.1111/jpn.12724 28503900

[22] Khajali F Wideman RF. Dietary arginine: metabolic, environmental, immunological and physiological interrelationships. Worlds Poult Sci J. (2010) 66:751–65. 10.1017/S0043933910000711

[23] Gao T Zhao MM Zhang L Li JL Zhou GH. Effects of in ovo feeding of l-arginine on the development of lymphoid organs and small intestinal immune barrier function in posthatch broilers. Anim Feed Sci Technol. (2017) 225:8–19. 10.1016/j.anifeedsci.2017.01.004

[24] Gao T Zhao M Zhang L Li J Yu L Gao F. In ovo feeding of l-arginine regulates intestinal barrier functions of posthatch broilers by activating the mTOR signaling pathway. J Sci Food Agric. (2018) 98:1416–25. 10.1002/jsfa.8609 28771730

[25] Yu LL Gao T Zhao MM Lv PA Zhang L Li JL. In ovo feeding of L-arginine alters energy metabolism in post-hatch broilers. Poult Sci. (2018) 97:140–8. 10.3382/ps/pex272 29077951

[26] Cheled-Shoval SL Amit-Romach E Barbakov M Uni Z. The effect of in ovo administration of mannan oligosaccharide on small intestine development during the pre- and posthatch periods in chickens. Poult Sci. (2011) 90:2301–10. 10.3382/ps.2011-01488 21934014

[27] Livak KJ Schmittgen TD. Analysis of relative gene expression data using real-time quantitative PCR and the 2(-Delta Delta C(T)) Method. Methods. (2001) 25:402–8. 10.1006/meth.2001.1262 11846609

[28] Bolyen E Rideout JR Dillon MR Bokulich NA Abnet CC Al-Ghalith GA. Reproducible, interactive, scalable and extensible microbiome data science using QIIME 2. Nat Biotechnol. (2019) 37:852–7. 10.1038/s41587-019-0209-9 31341288 PMC7015180

[29] Segata N Izard J Waldron L Gevers D Miropolsky L Garrett WS. Metagenomic biomarker discovery and explanation. Genome Biol. (2011) 12:R60. 10.1186/gb-2011-12-6-r60 21702898 PMC3218848

[30] Tahmasebi S Toghyani M. Effect of arginine and threonine administered in ovo on digestive organ developments and subsequent growth performance of broiler chickens. J Anim Physiol Anim Nutr. (2016) 100:947–56. 10.1111/jpn.12400 26608576

[31] Yuan C Ding Y He Q Azzam MM Lu JJ Zou XT. L-arginine upregulates the gene expression of target of rapamycin signaling pathway and stimulates protein synthesis in chicken intestinal epithelial cells. Poult Sci. (2015) 94:1043–51. 10.3382/ps/pev051 25771531

[32] Kim YS Ho SB. Intestinal goblet cells and mucins in health and disease: recent insights and progress. Curr Gastroenterol Rep. (2010) 12:319–30. 10.1007/s11894-010-0131-2 20703838 PMC2933006

[33] Mooradian AD Haas MJ. The effect of nutritional supplements on serum high-density lipoprotein cholesterol and apolipoprotein A-I. Am J Cardiovasc Drugs. (2014) 14:253–74. 10.1007/s40256-014-0068-1 24604774

[34] Aguiar Vallim TQ Tarling EJ Edwards PA. Pleiotropic roles of bile acids in metabolism. Cell Metab. (2013) 17:657–9. 10.1016/j.cmet.2013.03.013 23602448 PMC3654004

[35] Hermansen K Dinesen B Hoie LH Morgenstern E Gruenwald J. Effects of soy and other natural products on LDL:HDL ratio and other lipid parameters: a literature review. Adv Ther. (2003) 20:50–78. 10.1007/BF02850119 12772818

[36] Tangara M Chen W Xu J Huang FR Peng J. Effects of in ovo feeding of carbohydrates and arginine on hatchability, body weight, energy metabolism and perinatal growth in duck embryos and neonates. Br Poult Sci. (2010) 51:602–8. 10.1080/00071668.2010.520303 21058062

[37] Wu G. Amino acids: metabolism, functions, and nutrition. Amino Acids. (2009) 37:1–7. 10.1007/s00726-009-0269-0 19301095

[38] Seferovic MD Pace RM Carroll M Belfort B Major AM Chu DM. Visualization of microbes by 16S in situ hybridization in term and preterm placentas without intraamniotic infection. Am J Obstet Gynecol. (2019) 221:146.e141–6.e123. 10.1016/j.ajog.2019.04.036 31055031 PMC10357491

[39] Bi Y Tu Y Zhang N Wang S Zhang F Suen G. Multiomics analysis reveals the presence of a microbiome in the gut of fetal lambs. Gut. (2021) 70:853–64. 10.1136/gutjnl-2020-320951 33589511 PMC8040156

[40] Oakley BB Lillehoj HS Kogut MH Kim WK Maurer JJ Pedroso A. The chicken gastrointestinal microbiome. FEMS Microbiol Lett. (2014) 360:100–12. 10.1111/1574-6968.12608 25263745

[41] Polansky O Sekelova Z Faldynova M Sebkova A Sisak F Rychlik I. Important metabolic pathways and biological processes expressed by chicken cecal microbiota. Appl Environ Microbiol. (2015) 82:1569–76. 10.1128/AEM.03473-15 26712550 PMC4771310

[42] Layeghifard M Hwang DM Guttman DS. Disentangling interactions in the microbiome: a network perspective. Trends Microbiol. (2017) 25:217–28. 10.1016/j.tim.2016.11.008 27916383 PMC7172547

[43] Tang H Zhang N Ni H Xu X Liang Y. Increasing environmental filtering of diazotrophic communities with a decade of latitudinal soil transplantation. Soil Biol Biochem. (2021) 154:108119. 10.1016/j.soilbio.2020.108119

[44] De Oliveira JE Uni Z Ferket PR. Important metabolic pathways in poultry embryos prior to hatch. Worlds Poult Sci J. (2008) 64:488–499. 10.1017/S0043933908000160

[45] Uni Z Ferket PR Tako E Kedar O. In ovo feeding improves energy status of late-term chicken embryos. Poult Sci. (2005) 84:764–70. 10.1093/ps/84.5.764 15913189

[46] Rao C Coyte KZ Bainter W Geha RS Martin CR Rakoff-Nahoum S. Multi-kingdom ecological drivers of microbiota assembly in preterm infants. Nature. (2021) 591:633–8. 10.1038/s41586-021-03241-8 33627867 PMC7990694

[47] Graham EB Stegen JC. Dispersal-based microbial community assembly decreases biogeochemical function. Processes. (2017) 5:18. 10.3390/pr5040065

[48] Zelezniak A Andrejev S Ponomarova O Mende DR Bork P Patil KR. Metabolic dependencies drive species co-occurrence in diverse microbial communities. Proc Natl Acad Sci USA. (2015) 112:6449–54. 10.1073/pnas.1421834112 25941371 PMC4443341

[49] Schlatter DC Bakker MG Bradeen JM Kinkel LL. Plant community richness and microbial interactions structure bacterial communities in soil. Ecology. (2015) 96:134–42. 10.1890/13-1648.1 26236898

[50] Dai ZL Li XL Xi PB Zhang J Wu G Zhu WY. Regulatory role for L-arginine in the utilization of amino acids by pig small-intestinal bacteria. Amino Acids. (2012) 43:233–44. 10.1007/s00726-011-1067-z 21928075

[51] Ren W Chen S Yin J Duan J Li T Liu G. Dietary arginine supplementation of mice alters the microbial population and activates intestinal innate immunity. J Nutr. (2014) 144:988–95. 10.3945/jn.114.192120 24670969

[52] Abo H Chassaing B Harusato A Quiros M Brazil JC Ngo VL. Erythroid differentiation regulator-1 induced by microbiota in early life drives intestinal stem cell proliferation and regeneration. Nat Commun. (2020) 11:513. 10.1038/s41467-019-14258-z 31980634 PMC6981263

[53] Dai D Qiu K Zhang HJ Wu SG Han YM Wu YY. Organic acids as alternatives for antibiotic growth promoters alter the intestinal structure and microbiota and improve the growth performance in broilers. Front Microbiol. (2020) 11:618144. 10.3389/fmicb.2020.618144 33519778 PMC7840962

[54] Tamanai-Shacoori Z Smida I Bousarghin L Loreal O Meuric V Fong SB. Roseburia spp.: a marker of health? Future Microbiol. (2017) 12:157–70. 10.2217/fmb-2016-0130 28139139

[55] Lee YS Kim TY Kim Y Lee SH Kim S Kang SW. Microbiota-derived lactate accelerates intestinal stem-cell-mediated epithelial development. Cell Host Microbe. (2018) 24:833–46. 10.1016/j.chom.2018.11.002 30543778

[56] Tong X Xu J Lian F Yu X Zhao Y Xu L. Structural alteration of gut microbiota during the amelioration of human type 2 diabetes with hyperlipidemia by metformin and a traditional chinese herbal formula: a multicenter, randomized, open label clinical trial. mBio. (2018) 9:e02392-17. 10.1128/mBio.02392-17 29789365 PMC5964358

[57] Yang Y Zhang Y Xu Y Luo T Ge Y Jiang Y. Dietary methionine restriction improves the gut microbiota and reduces intestinal permeability and inflammation in high-fat-fed mice. Food Funct. (2019) 10:5952–68. 10.1039/C9FO00766K 31475718

[58] Song Y Wu MS Tao G Lu MW Lin J Huang JQ. Feruloylated oligosaccharides and ferulic acid alter gut microbiome to alleviate diabetic syndrome. Food Res Int. (2020) 137:109410. 10.1016/j.foodres.2020.109410 33233097

[59] Kimura I Ozawa K Inoue D Imamura T Kimura K Maeda T. The gut microbiota suppresses insulin-mediated fat accumulation via the short-chain fatty acid receptor GPR43. Nat Commun. (2013) 4:1829. 10.1038/ncomms2852 23652017 PMC3674247

[60] Murugesan S Nirmalkar K Hoyo-Vadillo C García-Espitia M Ramírez-Sánchez D García-Mena J. Gut microbiome production of short-chain fatty acids and obesity in children. Eur J Clin Microbiol Infect Dis. (2018) 37:621–625. 10.1007/s10096-017-3143-0 29196878

[61] Liu X Mao B Gu J Wu J Cui S Wang G. Blautia-a new functional genus with potential probiotic properties? Gut Microbes. (2021) 13:1–21. 10.1080/19490976.2021.1875796 33525961 PMC7872077

[62] Dehingia M Devi KT Talukdar NC Talukdar R Reddy N Mande SS. Gut bacterial diversity of the tribes of India and comparison with the worldwide data. Sci Rep. (2015) 5:18563. 10.1038/srep18563 26689136 PMC4686986

[63] Shen J Zhou J Xu Y Xiu Z. Prophages contribute to genome plasticity of Klebsiella pneumoniae and may involve the chromosomal integration of ARGs in CG258. Genomics. (2020) 112:998–1010. 10.1016/j.ygeno.2019.06.016 31220585

[64] Gao T Wang Z Dong Y Cao J Lin R Wang X. Role of melatonin in sleep deprivation-induced intestinal barrier dysfunction in mice. J Pineal Res. (2019) 67:e12574. 10.1111/jpi.12574 30929267

[65] Li CL Wang J Zhang HJ Wu SG Hui QR Yang CB. Intestinal morphologic and microbiota responses to dietary bacillus spp. in a broiler chicken model. Front Physiol. (2018) 9:1968. 10.3389/fphys.2018.01968 30705639 PMC6344408

[66] Wang WW Jia HJ Zhang HJ Wang J Lv HY Wu SG. Supplemental plant extracts from flos lonicerae in combination with baikal skullcap attenuate intestinal disruption and modulate gut microbiota in laying hens challenged by salmonella pullorum. Front Microbiol. (2019) 10:1681. 10.3389/fmicb.2019.01681 31396190 PMC6668501

[67] Kim S Shin YC Kim TY Kim Y Lee YS Lee SH. Mucin degrader Akkermansia muciniphila accelerates intestinal stem cell-mediated epithelial development. Gut Microbes. (2021) 13:1–20. 10.1080/19490976.2021.1892441 33678130 PMC7946046

[68] Grześkowiak Ł Grönlund MM Beckmann C Salminen S von Berg A Isolauri E. The impact of perinatal probiotic intervention on gut microbiota:double-blind placebo-controlled trials in Finland and Germany. Anaerobe. (2012) 18:7–13. 10.1016/j.anaerobe.2011.09.006 21979491

[69] O'Toole PW Marchesi JR Hill C. Next-generation probiotics: the spectrum from probiotics to live biotherapeutics. Nat Microbiol. (2017) 2:17057. 10.1038/nmicrobiol.2017.57 28440276

