# Supplementary material for: Supplemental L-Arginine Improves the Embryonic Intestine Development and Microbial Succession in a Chick Embryo Model
Source: Front Nutr. 2021 Oct 6;8:692305. doi: 10.3389/fnut.2021.692305 (PMC8526724; doi:10.3389/fnut.2021.692305)
Supplement: Supplementary file 1 [file Data_Sheet_1.docx]

***Supplementary Material***

| Genes | Primer sequence (5’-3’) | Accession no. |
| --- | --- | --- |
| *mTOR* | F: GGTGATGACCTTGCCAAACT | XM_417614.2 |
|  | R: CTCTTGTCATCGCAACCTCA |  |
| *S6K1* | F: CAATTTGCCTCCCTACCTCA | NM_001030721 |
|  | R: AAGGAGGTTCCACCTTTCGT |  |
| *4E-BP1* | F: GCGAATGTAGGTGAAGAAGAG | XM_424384.2 |
|  | R: AACAGGAAGGCACTCAAGG |  |
| *18S rRNA* | F: ATTCCGATAACGAACGAGACT | AF173612.1 |
|  | R: GGACATCTAAGGGCATCACA |  |

**Supplementary Table 1.** Sequences of real-time PCR primers


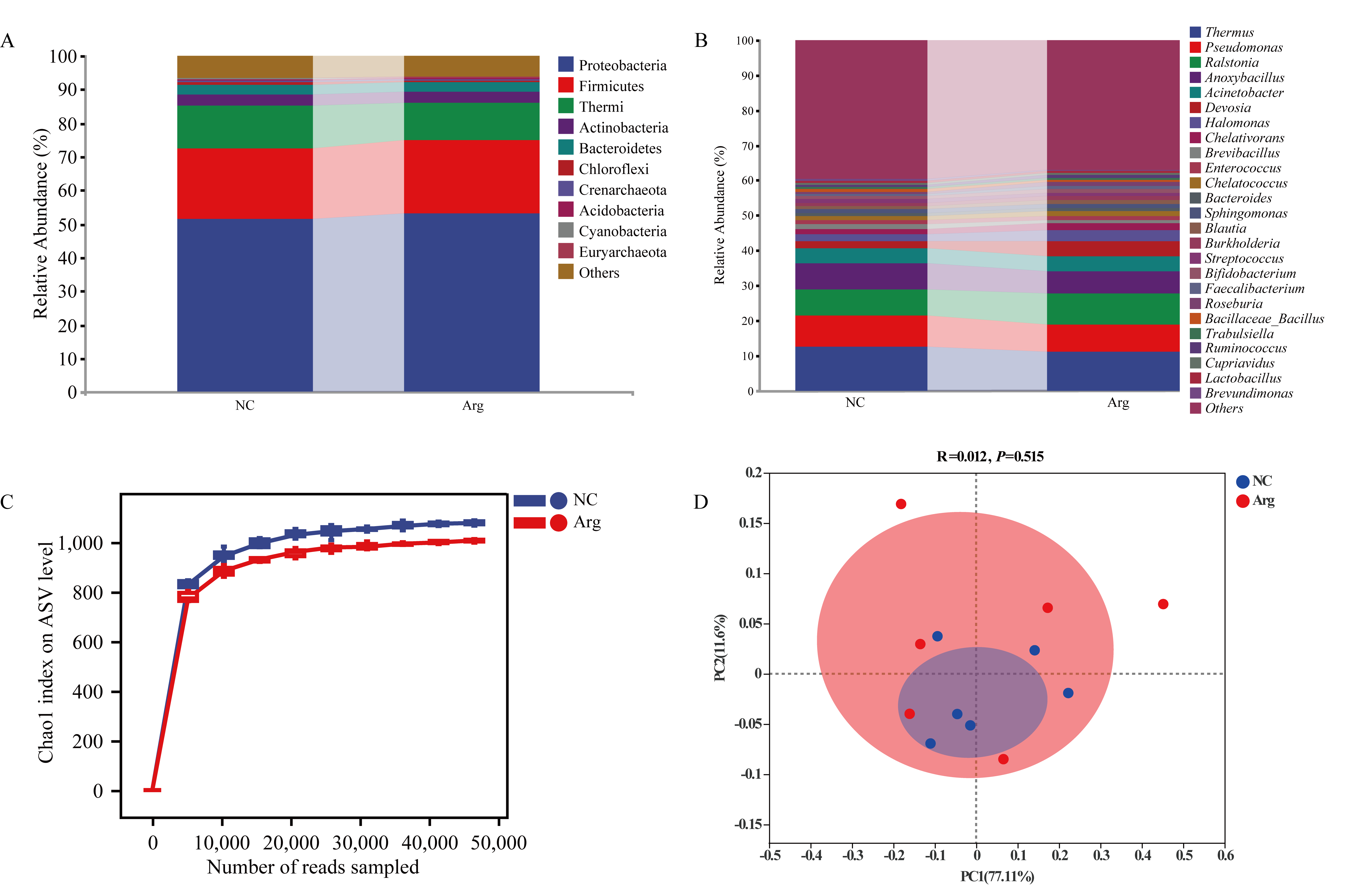


**Supplementary Figure 1.** Composition of intestinal microbiota and analysis of differences in microbiota of chicks at the age of embryos 19. **(A, B)** The composition of intestinal microbiota at phylum and genus level, respectively. **(C)** The Chao 1 index on ASVs level. **(D)** The principal coordinate analysis based on weighted unifrac distance. NC, non-injected control group; Arg, injected with 7 mg *L*-arginine group.
